# Understanding environmental influences on nutrition and physical activity behaviors: where should we look and what should we count?

**DOI:** 10.1186/1479-5868-3-33

**Published:** 2006-09-26

**Authors:** Kylie Ball, Anna F Timperio, David A Crawford

**Affiliations:** 1 Centre for Physical Activity and Nutrition Research, Deakin University, 221 Burwood Hwy, Burwood VIC 3125, Australia

## Abstract

Research interest in the influence of environmental factors on nutrition and physical activity behaviors has surged internationally in recent years. This is evident from a rapidly expanding literature and facilitated by advances in methodological and analytical approaches to assessing multiple levels of influence on health behaviors. However, a number of conceptual challenges complicate research endeavours in this field. The purpose of this paper is to provide a 'state of the science' overview of evidence regarding environmental influences on nutrition and physical activity behaviors. We focus particularly on a number of key conceptual and methodological issues, including: a consideration of how the environment is defined; the selection and operationalization of environmental exposures; and the importance of integrating existing understanding of individual influences on behavior with the emerging data on the role of the environment. We draw on examples from the published literature including our own research studies to illustrate these issues. We conclude by proposing a research agenda to progress understanding of the influences of the environment on population nutrition and physical activity behaviors.

## Background

Over the past decade there has been a growing recognition of the role of 'the environment' in influencing health and health behavior. For nutrition and physical activity behaviors, the emergence of the obesity pandemic has brought this issue into sharp focus. Numerous reviews and reports by expert bodies throughout the world highlight the importance of 'environmental' factors on obesity and obesity-risk behaviors, and in terms of obesity prevention, there have been strong calls to focus on changing the environment [1-3]. The notion of creating supportive environments for health is certainly not new. John Snow's actions to disable the Broad Street pump in order to stem the spread of the cholera epidemic in London in 1854 [4] is the classic example of an environmental intervention to protect health. With regards to obesity, Rimm and White drew attention to the central role of 'the environment' more than 25 years ago when they argued that population obesity was a "product of our environment" [5]. The re-emergence of social ecological theory [6,7] and its application to the study of nutrition and physical activity behaviors [8,9] provides a recent example of the shift in focus from individual-level (psycho-social) influences on health to environmental influences.

While much has been written about the potential impact of the environment on nutrition and physical activity behaviors, and in relation to obesity risk, existing empirical evidence regarding the importance of environmental factors is at best patchy, with many important nutrition and physical activity behaviors (e.g., soft drink consumption; children's active free play outside of school; sedentary behaviors in the home) having not been examined. While a growing body of research on utilitarian walking trips is emerging from the urban, planning and transport literatures [10], much of the published literature examining the environment and physical activity has been concerned with recreational walking among adults [11-18]. Further, the existing research has tended to focus on only a limited range of explanatory variables within the physical environment, with little attention having been paid to variables relevant to the social, cultural and policy environments. The study of environmental influences on nutrition and physical activity behaviors is a relatively new science, and at this point it is far from clear where we should look and what we should count.

## Discussion

### What do we mean by 'environment'?

To paraphrase a leading international expert in the analysis of environmental effects on health, identifying "true" environmental differences requires identifying "true" environments [19]. While intuitively this seems like simple logic, in practice identifying 'true' environments that may exert influence on nutrition and physical activity behaviors may not be straightforward for a number of reasons. Firstly, most people live and function in multiple contexts or settings [7], all of which are likely to have some influence on their nutrition and physical activity behaviors. Among children, for example, there is a growing literature documenting the role of the family environment in influencing these behaviors [20-22]. However, children also spend a considerable amount of time at school, and an increasing number of studies have examined how the school environment might influence nutrition and physical activity behaviors both at school and more broadly [23-25]. Similarly, the environments in which adults work or study may influence their nutrition and physical activity behaviors [9,26]. More recently, there has been an increasing focus in the literature on the 'neighbourhood' environments in which people live as another source of influence [10,11,13,27-48].

A second difficulty in attempting to define environment in health behavior studies is that people live and work in multiple geographic areas, some of which are nested within others, such as streets within census or postal areas; neighbourhoods within larger areas like cities, states, regions counties or countries; and potentially even larger geographic areas such as international trading blocks, or developed versus developing countries. Furthermore, some people may share the same residential neighbourhood and workplace, while others may live in the same neighbourhood but work in different places of employment, or vice versa. Defining a true 'environment' is therefore challenging. While some studies have focused on one environmental context, there is a need to consider the relative influences of different environments that may or may not be nested within one another, yet few studies to date have attempted to do so. Jeffery et al. [31], for example, reports on one of the only studies to examine environmental attributes in both the residential neighbourhood and the environment surrounding people's workplaces, finding that access to fast food restaurants was differentially associated with fast food consumption and body mass index, dependent on whether access was defined as proximity to home or to work.

A third factor that is relevant to a discussion about the definition of environments in health behavior studies relates to the fact that people not only live in multiple contexts and geographic environments, but there are also different types of environmental influences operating across these domains. These include the physical environment, comprising both the built and natural environments, and also the social, cultural and policy environments [6]. Many studies of environmental influences on nutrition and physical activity behaviors have focused on the physical environment. Fewer have examined the family environment or the social or cultural environment within local neighbourhoods, and fewer still have examined the relative contributions of different types of environment [13,30]. However, nutrition and physical activity behaviors are likely to be influenced by all these. For example, it is likely that proximal factors such as having a partner or children who dislike vegetables, intermediate factors such as access to quality fresh produce locally, and very distal factors such as legislation governing food taxes, all have some impact on an individual's food choice.

### Defining a 'neighbourhood'

Many existing studies of 'neighbourhood environment' influences have used administrative definitions of neighbourhoods, such as census-defined tracts, or postal codes [e.g., [28,30,34]]. One probable reason for this is that data on many of the indicators studied (eg. SES of areas, crime, poverty, facilities) are already readily available at these levels. However, administratively-defined neighbourhoods may not be consistent with resident perceptions of their neighbourhood. Coulton and colleagues [37], for example, compared boundary maps drawn by residents to census-defined block groups and found differences in both the areas mapped and social indicators between the resident and administrative boundary maps. Furthermore, there were also inconsistencies between resident-drawn maps. Behavioral mapping among children also suggests that perceptions of the neighbourhood environment are highly individual [27,38]. It is possible that reliance on administrative boundaries may underestimate the effects of the neighbourhood environment on nutrition and physical activity behaviors because real conditions affecting residents may not be adequately captured. Likewise, matching of objective features of an environment and perceptions of the environment is likely to be problematic.

An alternative approach is to examine neighbourhood environments specific to individuals, such as the environment within a 400 m radius of an individual's residential address. In this approach each individual is assigned a unique neighbourhood. However, as outlined by Giles-Corti et al. [39], there is little agreement in the literature as to what might constitute an appropriate distance or boundary from home (or work or school), and to date multiple definitions have been applied (e.g., 400 m [40,41]; 800 m [41]; 1 km [42]; 0.5 miles [43]; 2 miles [31]; 10–15 mins' walk [10,44]; and 10 miles, or 20 mins' drive [43]. The choice of an appropriate distance to define a 'neighbourhood' may be dependent on a number of considerations. These include the behavior of interest and the likelihood of this behavior occurring close to home [39]; policy or planning guidelines that might stipulate, for instance, that each resident should have a park or transit stop within a particular distance from home; or the extent of children's independent mobility and what constitutes an appropriate walking distance for children [36]. For example, Giles-Corti et al. [39] propose that a neighbourhood environment defined as between 10 and 15 minutes' walk from home is appropriate for examining recreational walking, since this is consistent with current physical activity recommendations of 30 minutes' moderate-intensity activity daily. However, such a definition is likely to be inappropriate in studies of other physical activity behaviors, of nutrition-related behaviors, or in research involving other target groups (e.g. young children). Although different definitions of what constitutes a neighbourhood may be appropriate for different population groups or behaviors of interest, making comparisons between studies that have applied different definitions is difficult.

Defining unique neighbourhoods for each participant is advantageous in providing increased specificity. However, these individualized definitions of neighbourhood do not geographically align with existing administrative definitions, and hence the collection of neighbourhood attribute data in this approach can be time- and labour-intensive, as the data of interest are not likely to exist at this level of specificity. Although it may be easier to collect data for smaller neighbourhood areas, there is also a chance that key environmental factors located just outside this arbitrary boundary may be missed. To further complicate this issue, access to vehicles or public transport may mean that people are able to, and may prefer to, access a larger geographic area than is determined a priori as being within their neighbourhood boundaries. For example, parents of young children have reported that they are willing to drive long distances to take their children to good quality parks [38]. Similarly, adults may access facilities or food stores en route to other places they regularly travel to by car (e.g., en route to their workplace or child's school).

### Which aspects of the environmental should we focus on?

In addition to decisions regarding the definition of environment, the investigation of environmental determinants of nutrition and physical activity behaviors requires careful consideration of the selection and operationalization of exposures of potential importance. Literally thousands of environmental factors might influence nutrition and physical activity behaviors. Which of these exposures should be a focus, and what particular characteristics of these exposures should be examined? For example, if we consider a single physical activity facility – a walking trail – is it the existence of a trail; its length; its proximity to one's home; its quality or level of maintenance; the aesthetic characteristics or the safety of the areas though which the trail passes; the steepness of any inclines along the trail; its frequency of use by others; the availability of washrooms or drinking fountains along the trail; or other characteristics that are important? Similarly, if we take a single nutrition-related exposure as an example – a fruit and vegetable store – should we focus on the distance from a resident's home; its accessibility by car, public transport or by foot; the quality, range or cost of produce; opening hours; standard of service; availability of competing stores; familial preferences for the produce served; or other characteristics? While most research in this area to date has focused primarily on the availability of facilities, clearly a multitude of factors may be important. For instance, we have found that convenience of facilities locally was an important correlate of walking for exercise/recreation, but so too were the perceived aesthetics and attractiveness of the local neighbourhood [11]. Similarly, attractiveness of public open space has been found to be associated with higher rates of walking [14]. Availability of fast food outlets and vending machines in schools may be less important than policies governing students' access to these facilities at different times of the school day [49].

What are the implications of this array of exposures for a researcher trying to investigate environmental influences on nutrition and physical activity behaviors? Clearly it is not possible to examine all potentially important exposures in a single study (or perhaps a lifetime of research). How then does one select and prioritize those exposures to investigate? Unfortunately, many studies fail to provide clear justification for the particular exposures selected, and there appears a tendency for studies to be guided more by the data that are available than by careful a priori theoretical selection and conceptualization of key environmental exposures. Admittedly, collection of new environmental data, particularly in large population-based studies, is time- and labour-intensive and thus costly. Nonetheless, a reliance on existing data simply because it is available can be problematic for a number of reasons. Aside from logistical issues related to, for instance, variable data quality and completeness, not all environmental variables are associated with nutrition and physical activity behaviors, and sometimes even those we may expect to be important are not. For instance, in their review of the literature, Humpel et al. [33] demonstrated that across studies, weather, heavy traffic, and various safety indices were not consistently related to physical activity. The reasons for these consistently null associations are not clear: the measures used may have been inadequate to detect associations, or it may be that associations hold for some target groups but not others (further discussion of potential 'moderating' characteristics is provided below). However, it may also be the case that there is no association of behavior with certain environmental constructs. In other words, simply because an exposure exists does not mean it will be important. This in itself is not problematic, since null findings can be very informative. However, investigating associations of environmental factors with health behaviors without a plausible conceptual rationale for doing so may well result in null findings that are not surprising, nor informative.

Accepting the requirement for careful selection and conceptualization of exposures, several other factors require consideration. A growing body of evidence suggests that associations of specific environmental factors with physical activity vary according to the particular behavioral outcomes being studied [33,39,40,45]; the case is likely to be the same for nutrition-related behavioral outcomes [46]. For instance, coastal proximity and access to fresh seafood markets may be important for promoting fresh fish consumption but have no impact on fruit and vegetable intake, and only a weak impact on 'overall' dietary patterns. Similarly, living in an area with an abundance of leisure centres, swimming pools, tennis courts and bowling greens might impact participation in those specific physical activities, but have no effect on walking or cycling, and only a weak effect on overall physical activity. There is also evidence that different environmental factors are likely to be differentially associated with different types of walking. In our recent study of Socioeconomic Status and Activity in Women (SESAW) [47], we found that the accessibility of walking tracks was predictive of walking for exercise, but not for transport, whereas the reverse was true for street connectivity. Considering the theoretical links between environmental exposures and specific behavioral outcomes is clearly critical and has been a focus of several recent reviews [39,45]. This is likely to be facilitated by the development of new measures to capture behaviors occurring specifically within the environmental boundaries under study, such as walking for transport within one's own local neighbourhood [44].

Also of importance when selecting environmental exposures for study is a consideration of the characteristics of the target group. Those characteristics of the environment that predict nutrition and physical activity behaviors are likely to vary according to the sex, age, race, and other characteristics of the individual or target group of focus. For instance, our own research with women [47] and children [38] suggests that a highly connected street design is optimal for increased walking among adults, but this is not the case for children – rather, streets that are cul-de-sacs or courts – dead-ends rather than interconnectivity – are more important for this population group. Kremers and colleagues [50] have recently proposed a conceptual framework that suggests that the importance of environmental influences on nutrition and physical activity-related behaviors might vary according to demographic and other target group characteristics, including personality, health habits, and awareness of and involvement in personal health behaviors. Application of this framework and testing of such hypotheses is required.

Beyond selection, there is also a need to carefully operationalize environmental constructs. How do we assess these constructs? Here again there are many issues to be dealt with, including ensuring the validity and reliability of indices; the use of perceived versus objective assessment [51]; and the timing of measurement of exposures (eg how long does it take for environmental factors to get 'under the skin'?). The field is further complicated by the possibility that environmental measures, and potentially results, may be country-specific – for instance, we don't yet know whether constructs such as 'street connectivity' have precisely the same meaning in the US, Australia, Europe and Asia. It is promising to see emerging studies of reliability/validity testing of perceived and objectively assessed environmental measures [48,52], but much work on assessment of environmental exposures and their relationships with behavior patterns across different countries remains to be done.

In summary, these issues highlight the importance of careful consideration of the definition of the environment, and the selection and operationalization of particular exposures. These should be guided by the particular study hypotheses; the exposures and outcome behaviors of interest; and the population group being investigated. Importantly, our definition of 'environment', and our selection of particular environmental exposure variables, should be based on sound theoretical considerations. That is, it is important to clarify which *specific *features of the social, physical and policy environment we hypothesize will influence which specific nutrition and physical activity behaviors, and how they might do so? This may well result in a need for measures not only of potential environmental influences, but also of intrapersonal factors that might mediate the influence of the environment on health behaviors, as described below.

### How do we integrate understanding of environmental and other influences?

Emerging research supports the notion that particular aspects of the environment influence nutrition and physical activity behaviors among adults and children. Although there is little doubt that environments play an important role, in our enthusiasm to embrace new paradigms focused on environmental determinants, it is important to remember that decades of past research have identified a vast range of intrapersonal determinants of nutrition and physical activity behaviors, including motivation, self-efficacy, knowledge, perceived barriers, intentions, preferences, attitudes and beliefs [53-55]. How do we go about integrating our existing understanding of these intrapersonal influences with emerging evidence attesting to the importance of broader social and physical environments?

The examination of behavioral determinants from multiple levels simultaneously has the potential to provide much insight into the *relative *importance of personal, social and physical environmental influences on nutrition and physical activity behaviors. To date few studies have looked at multiple determinants concurrently. Two recent exceptions in the physical activity literature reported quite similar patterns of findings – that selected personal, social, and physical environmental factors were all associated with leisure-time walking [47] and with exercising as recommended [56], with personal influences showing the strongest associations. In separate analyses of data from the SESAW study examining personal, social and environmental determinants of women's fruit and vegetable consumption [57] personal and social factors were found to be more important correlates of consumption than were the environmental factors studied (availability of fruit and vegetable outlets and supermarkets). Clearly, however, additional research is required to confirm such findings and to investigate a broader range of potential determinants from across the levels of influence. In addition, little is known about the interactions amongst personal, social and environmental factors. For instance, what are the dietary ramifications for an individual who has high self-efficacy for increasing fruit intake, but who lives and works in a neighbourhood with no access to fresh fruit? What about an individual who has low self-efficacy but lives in close proximity to several fresh fruit stores and markets? The potential combinations of specific behavioral influences and their interactions from multiple levels appear limitless.

How then do we integrate our understanding of these different levels of influence? Fortunately we are assisted in our efforts with the advancement of appropriate methodologies, in particular multilevel statistical techniques that enable the analysis not only of the relative contribution of different levels (individual, social, environmental) of determinants, but also of their (cross-level) interactions. However, it is of concern that the advancement of analytical methods and their application has raced ahead of the conceptual or theoretical frameworks needed to advance understanding in this field. That is, while guiding frameworks such as Social Ecological Theory are useful for considering behavioral determinants broadly, we currently lack strong and well-articulated conceptual models for theorizing about and testing the specific mechanisms by which particular environmental exposures might interact with individual factors to influence health behaviors. Only very recently has a potential model – termed the 'dual-process view' of environmental determinants of energy-balance-related behaviors [50] – been articulated. According to this model, environmental factors influence behavior both directly, but also indirectly, via a number of hypothesized mediating pathways. While this is to be commended as an excellent first step, much work remains in applying the model to test its usefulness in delineating the causal pathways linking environmental and individual behavioral determinants. In summary, the lack of explicit evidence-based theoretical models is arguably the major limitation of work in this field to date and perhaps the greatest challenge we face in advancing research in this area.

## Conclusion

The recent enthusiasm internationally for investigating the impact of the environment on health augurs well for efforts to broaden and advance our understanding of the determinants of nutrition and physical activity behaviors. Such research has a range of important practical and policy implications. "One size fits all" blanket approaches to promoting nutrition and physical activity assume that 'background' components are equal, whereas a growing body of research demonstrates that people live in a range of environments, not all of which are conducive to healthy eating and physical activity. In addition to informing environmental planning and policy efforts, knowledge of key environmental determinants may be vital in information and awareness-raising approaches to promoting healthy behavior, for example by helping people to make the most of the environments in which they live, work and play. Findings may also inform community advocacy efforts. If residents are aware that traffic calming, green spaces or fresh fruit availability are important, for instance, they can lobby to have these elements of their neighbourhoods modified.

What are the implications for research? There is little consensus as to the most appropriate approaches for defining environments or the selections of environmental exposures that might be important for nutrition and physical activity behaviors. Much research has been opportunistic, focussing on existing measures of facility availability only. Some researchers have attempted to develop composite indices taking into account that accessibility of facilities depends not only on distance but also attractiveness, importance to the user, and so forth [39], and this developing field of work might address some of these complexities. With few exceptions [58-61], much research in the field to date has also been observational, which does not provide strong evidence as to the temporal or causal nature of associations of environmental factors with behavior.

In our opinion, however, the most significant challenge for researchers in this field is to clearly articulate theoretical models from which to develop testable hypotheses regarding the influence of environmental exposures on nutrition and physical activity behaviors. Conceptual development currently lags behind analytical advances, and there remains considerable complexity and little evidence to guide most appropriate definitions of environment, or identification of important influences on behavior. There remains a critical need for continued development and application of theoretical models linking environmental and individual exposures to health behaviors.

### Future research: a proposed agenda

Based on the overview of the literature and the methodological and conceptual challenges outlined here, we propose the following key priorities as part of a research agenda for advancing our understanding of environmental determinants of nutrition and physical activity behaviors.

Future research should:

• Focus strongly on progressing development and testing of clear conceptual models. Hypothesize specific, testable hypotheses that explicitly link different levels of influence from social ecological models to context-specific nutrition or physical activity behavioral outcomes in particular population groups; and test these models with appropriate empirical data (which may require new data collections): for example, a model of adults' leisure-time walking; of children's active free play; of adolescents' sport participation; of adolescents' soft drink consumption; or of older adults' fruit and vegetable consumption.

• Consider the relative influence of factors within different environments in which people live and function (home, work, school, neighbourhood).

• Focus on progressing methodological efforts, including refining environmental measures. Further develop, reliability and validity test environmental audit instruments. Investigate the implications of using different sized-buffer zones for different environmental exposures, behaviors and across different target groups.

• Incorporate less well-studied nutrition and physical activity behaviors (eg other than walking).

• Capitalize on available analytical techniques to test theoretically-based hypotheses regarding the cross-level interactions of individual, social and environmental determinants, and mediating and moderating relationships amongst determinants from different levels of analysis.

• Progress conceptual understanding and empirical evidence regarding the role of the social, cultural and policy environments.

• Extend primarily observational research studies, for example, by utilizing opportunities for 'natural experiments' such as assessing the impact of new food stores or physical activity facilities

There seems little doubt that aspects of 'the environment' have a potent influence on nutrition and physical activity behaviors. As yet, research in this area is in its infancy. Much remains to be done, creating exciting opportunities for innovative methodological, conceptual and empirical research in the coming years.

## Declaration of competing interests

The author(s) declare that they have no competing interests.

## Authors' contributions

KB conceived of and drafted the first outline of the paper. Each author wrote at least one key section of the manuscript. All authors read and approved the final manuscript.
